# Digital PCR for the Analysis of *MYC* Copy Number Variation in Lung Cancer

**DOI:** 10.1155/2020/4176376

**Published:** 2020-09-19

**Authors:** Alexander Brik, Daniel G. Weber, Swaantje Casjens, Peter Rozynek, Swetlana Meier, Thomas Behrens, Georgios Stamatis, Kaid Darwiche, Dirk Theegarten, Thomas Brüning, Georg Johnen

**Affiliations:** ^1^Institute for Prevention and Occupational Medicine of the German Social Accident Insurance, Institute of the Ruhr University Bochum (IPA), Bochum 44789, Germany; ^2^Department of Thoracic Surgery, University Medicine Essen, Essen 45239, Germany; ^3^Department of Interventional Pneumology, University Medicine Essen, Essen 45239, Germany; ^4^Department of Pathology, University Medicine Essen, Essen 45239, Germany

## Abstract

**Background:**

*MYC* (v-myc avian myelocytomatosis viral oncogene homolog) is one of the most frequently amplified genes in lung tumors. For the analysis of gene copy number variations, dPCR (digital PCR) is an appropriate tool. The aim of our study was the assessment of dPCR for the detection of *MYC* copy number variations (CNV) in lung tissue considering clinicopathological parameters. *Material and Methods*. *MYC* status was analyzed with dPCR as well as qPCR (quantitative PCR) using gDNA (genomic DNA) from tumor and adjacent nontumor tissue samples of lung cancer patients. The performance of *MYC* was estimated based on the AUC (area under curve).

**Results:**

The results of the *MYC* amplification correlated significantly between dPCR and qPCR (*r*_S_ = 0.81, *P* < 0.0001). The *MYC* copy number revealed by dPCR showed statistically significant differences between tumor and adjacent nontumor tissues. For discrimination, a sensitivity of 43% and a specificity of 99% were calculated, representing 55 true-positive and one false-positive tests. No statistically significant differences could be observed for age, sex, and smoking status or the clinicopathological parameters (histological subtype, grade, and stage).

**Conclusion:**

The results of the study show that dPCR is an accurate and reliable method for the determination of *MYC* copy numbers. The application is characterized by high specificity and moderate sensitivity. *MYC* amplification is a common event in lung cancer patients, and it is indicated that the determination of the *MYC* status might be useful in clinical diagnostics.

## 1. Introduction

Lung cancer shows the highest incidence and mortality worldwide with nearly 2.1 million cases and 1.8 million deaths in 2018, respectively [1]. The most common histological type of lung cancer is non-small-cell lung carcinomas (NSCLC) accounting for 85% of all lung cancer cases [2]. Among other changes, lung cancer is marked by genomic instability resulting in a high frequency of somatic mutations and extensive genomic alterations in individual genomes [3]. Copy number variation (CNV) designates an alteration of genomic DNA characterized by a change of DNA sequence numbers in the normal (diploid) genome. These DNA alterations may affect individual genes, chromosomal regions, or whole chromosomes. It was shown that CNVs are connected with lung cancer as well as several other malignant diseases [4, 5]. Generally, in cancer, a decrease or increase of DNA copy numbers might affect tumor suppressor genes and oncogenes, respectively.

One of the most frequently amplified genes in lung tumors is v-myc avian myelocytomatosis viral oncogene homolog (*MYC*) [6]*. MYC* is located on chromosome 8q24.21 and codes for a transcription factor playing a role in cell cycle progression, apoptosis, and cellular transformation. Using the “The Cancer Genome Atlas” (TCGA) dataset, Schaub et al. showed that *MYC* is amplified in 21% of approximately 9,000 samples across 33 different cancers [7]. Particularly, in lung squamous cell carcinoma, *MYC* is amplified in 37% of the samples. Thus, *MYC* alteration might be a promising biomarker candidate for the detection of lung cancer in tissues. Additionally, several studies demonstrated that *MYC* amplification is associated with poor prognosis in NSCLC and small-cell lung cancer (SCLC) [8, 9]. However, an increase of *MYC* gene copies positively affects the response to therapies as seen after treatment of NSCLC by tyrosine kinase inhibitors (TKIs) [10]. Currently, the development of techniques targeting *MYC* for cancer therapy is being comprehensively investigated [11, 12].

Digital PCR (dPCR) was introduced as a method for precise copy number detection [13]. In general, dPCR is a sensitive, robust, and accurate tool for the detection of low copy targets and rare alleles. In contrast to quantitative PCR (qPCR), dPCR is marked by higher tolerance to enzyme-inhibiting substances, higher precision, higher sensitivity, and improved reproducibility [14–17]. Notably, there is no need for a standard curve or calibrator sample. Thus, dPCR might be an accurate tool for the application in clinical diagnostics [18].

The aim of our study was the assessment of dPCR for the detection of *MYC* CNV in primary lung cancer tissue and adjacent nontumor tissues. Additionally, the *MYC* copy number status was analyzed considering clinicopathological parameters of the lung cancer patients.

## 2. Material and Methods

### 2.1. Ethics Statement

All participants of the study provided informed consent. The study was designed according to the rules guarding patient privacy and with the approval from the ethics committee of Faculty of Medicine, Ruhr University Bochum (registration number 4552-12).

### 2.2. Study Population

Between 03/2013 and 06/2015, 656 subjects with lung cancer, mesothelioma, or other benign diseases of the respiratory system were recruited in the Ruhrlandklinik (Essen, Germany). Within the context of surgical interventions, tissue samples were collected from 241 lung cancer patients.

### 2.3. Sample Preparation

Removed tissue samples were immediately cooled to 4°C and washed with isotonic saline. After pathological evaluation, tissue samples were stored at −80°C. For the determination of *MYC*, tissue samples (tumor and adjacent nontumor tissues without signs of inflammation) from 129 lung cancer patients were used.

### 2.4. Detection of CNV

Two 40 *μ*m sections of frozen tissue were used for isolation of genomic DNA (gDNA). To each tissue sample, 80 *μ*L Proteinase K (Qiagen, Hilden, Germany) was added and homogenized for 1 minute with 850–1000 rpm using a Schuett Homgen plus (Schuett Biotec GmbH, Göttingen, Germany). Then, 320 *μ*L Buffer ATL (Qiagen) was added and incubated over night at 56°C with 1,000 rpm using a ThermoMixer (Eppendorf, Hamburg, Germany). Isolation of gDNA was performed using the QIAamp DNA Mini Kit (Qiagen) according to the manufacturer's instructions. Volumes of all reagents were adapted proportionally with increasing template quantity. Quantification of gDNA was performed using a Qubit Fluorometer and Qubit dsDNA BR Assay Kits (Thermo Fisher Scientific, Darmstadt, Germany).

Digestion of 400 ng gDNA was carried out in 16 *μ*L Reaction Mix using 10 U EcoRI (BioLabs, Frankfurt am Main, Germany) and 10× EcoRI buffer (BioLabs) for 1 hour at 37°C followed for 10 minutes at 65°C. Preamplification was performed using a MJ Research PTC-200 Thermal Cycler (Bio-Rad Laboratories, Hercules, CA) with 15 ng gDNA as template in 10 *μ*L Reaction Mix including 1× PreAmp Master Mix (Thermo Fisher Scientific), 0.01× target assay, and 0.01× reference assay using the following conditions: 95°C for 10 minutes, followed by 5 cycles at 95°C for 15 seconds, and 60°C for 2 minutes.

qPCR was performed using a 7900HT Real-Time PCR System (Thermo Fisher Scientific) with 10 ng gDNA as template as described previously by Mayo et al. [19]. All reactions were performed in triplicates, and nontemplate controls were included in all assays. As a calibrator, 10 ng of human genomic DNA (product code 11691112001; Roche, Mannheim, Germany) was used. CopyCaller v2.0 (Thermo Fisher Scientific) was used for data analysis. The standard deviation of the sample replicates was <0.13, the *z*‐score < 2.1, and the Ct (cycle threshold) of the reference gene was <32.

dPCR was carried out using a Dual Flat Block GeneAmp PCR System 9700 (Thermo Fisher Scientific) and QuantStudio 3D Digital PCR 20K Chip Kits v2 (Thermo Fisher Scientific) with 35 ng gDNA as template according to the manufacturer's instructions [20], aiming at a concentration of target and reference between 200 and 2,000 copies/*μ*L. All reactions were performed in duplicates, and nontemplate controls were included. Copy numbers were determined using a QuantStudio 3D Digital PCR Instrument (Thermo Fisher Scientific) and analyzed using the QuantStudio 3D Digital PCR Software version 3.0 (Thermo Fisher Scientific). *MYC* copy number/nucleus was calculated as 2× ratio of *MYC* copy number to *RNase P* copy number.

Both qPCR and dPCR were performed using the FAM-labeled *MYC* TaqMan Copy Number Assay (product code Hs02758348_cn; Thermo Fisher Scientific) and the VIC-labeled *RNase P* TaqMan Copy Number Reference Assay (product code 4403326; Thermo Fisher Scientific) as reference. Copy numbers of *MYC* and *RNase P* in a sample were measured in parallel using a single chip.

### 2.5. Statistical Analyses

Statistical analyses were performed using SAS software, version 9.4 (SAS Institute Inc., Cary, NC, USA). The agreement between *MYC* copy number statuses via different methods was analyzed by Spearman's correlation coefficient and illustrated using Bland-Altman plots. In a Bland-Altman plot, the arithmetic mean of two markers (*x* axis) is plotted against the difference between the markers (*y* axis). The horizontal line describes the mean of the differences, and the dashed lines describe the 95% confidence interval (CI) assessed as the mean difference ± 1.96 standard deviation (SD). Dot plots with median and interquartile range (IQR) were used to depict the distribution of single biomarkers. The Mann-Whitney *U* tests were applied to examine group differences, and the Wilcoxon signed-rank tests were applied to compare two related samples. *P* values < 0.05 were considered as statistically significant. Receiver operating characteristic (ROC) curves were used to quantify classification performance of the biomarkers. The accuracy of the diagnostic tests was depicted by the area under curve (AUC) and its 95% CI. Biomarker cut-offs were determined using fixed false positive rates (FPRs), maximum Youden's Index (YI), and set as mean of the biomarker concentration in nontumor tissues plus twofold or threefold SD. The chi-squared test was used to compare *MYC* status and clinicopathological parameters.

## 3. Results

### 3.1. Study Population

The clinicopathologic parameters of the 129 lung cancer patients are summarized in Table 1. Median age of the patients was 67 years, and 50.7% of the patients were younger than 67 years. The majority of the patients (62.0%) were males. More than half of the participants (54.3%) were former smokers, whereas 34.1% were current smokers and 11.6% were nonsmokers. Mostly, patients were diagnosed with adenocarcinoma (51.9%) or squamous cell carcinoma (23.3%). The majority of the patients (83.0%) were diagnosed with tumor stages T1 and T2.

### 3.2. DNA Treatment Prior to PCR

**Figure 1:**
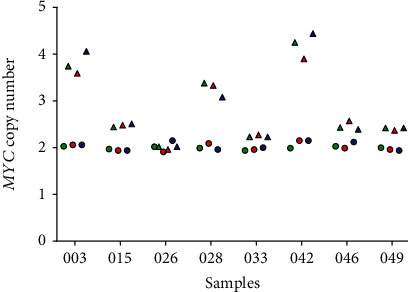
*MYC* copy numbers of samples without and with pretreatment prior to PCR (green: no gDNA pretreatment; red: gDNA digestion with EcoRI; blue: preamplification of gDNA; triangle: tumor samples; circle: nontumor samples).

In order to assess the possible influence of DNA pretreatment, eight sample pairs (consisting of tumor and nontumor gDNA) were either digested, preamplified, or not treated prior to PCR. As shown in Figure 1, the revealed *MYC* copy numbers of the digested, preamplified, and nontreated gDNA samples were similar and the differences due to the pretreatment were marginal. Notably, in samples with *MYC*copy numbers > 3, slightly higher differences between the different pretreatments could be observed. The raw data of the dPCR experiments are presented in Table 1 of the Additional File 1.

### 3.3. qPCR vs. dPCR

For the methodological comparison of the performance of dPCR and qPCR, 116 tumor and nontumor samples from 58 patients were analyzed. Based on the previously obtained results, samples were not treated prior to dPCR and qPCR. The raw data of the qPCR experiments are presented in Table 2 and of the dPCR experiments in Table 3 of the Additional File 1.

**Figure 2:**
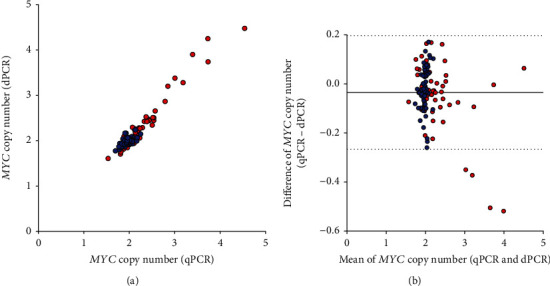
(a) Correlation of *MYC* copy numbers in 116 samples (tumor and adjacent nontumor tissue) from 58 lung cancer patients assessed by qPCR and dPCR (red: tumor tissue; blue: adjacent nontumor tissue). (b) Bland-Altman plot of *MYC* copy numbers assessed by qPCR and dPCR in 116 samples (tumor and adjacent nontumor tissue) from 58 lung cancer patients (red: tumor tissue; blue: adjacent nontumor tissue). The horizontal solid line indicates the mean difference at -0.03, the horizontal dashed lines indicate the range of ±1.96 × SD (upper limit = 0.20; lower limit = −0.27).

As shown in Figure 2(a), results of *MYC* amplification correlated significantly between dPCR and qPCR for all analyzed groups: tumor and nontumor samples (*r*_S_ = 0.81, *P* < 0.0001), tumor samples (*r*_S_ = 0.93, *P* < 0.0001), and nontumor samples (*r*_S_ = 0.54, *P* < 0.0001). Additionally, the Bland-Altman plot indicated a good overall agreement between dPCR and qPCR in the corresponding samples (Figure 2(b)), i.e., 96.55% of the measurements fitted within the limit of agreement.

### 3.4. Assessing of *MYC* Copy Number by dPCR

**Figure 3:**
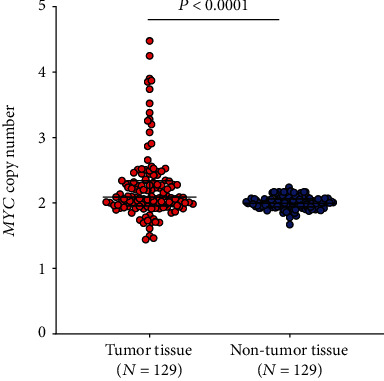
Distribution of *MYC* copy numbers in tumor and adjacent nontumor tissues from 129 patients. The Mann-Whitney *U* test was performed to examine group differences. Vertical bars indicate median and interquartile ranges.

For the assessment of *MYC* alteration in lung cancer, *MYC* copy numbers were determined in tumor and adjacent nontumor tissue of 129 lung cancer patients using dPCR. The raw data of the dPCR experiments are presented in Table 3 of the Additional File 1. As indicated, no treatment was performed prior to dPCR. The median *MYC* copy number in tumor samples was 2.09 (IQR: 1.96–2.34) and in nontumor samples 1.99 (IQR: 1.94–2.06). The mean *MYC* copy number in tumor samples was 2.25 with an SD of 0.53 and in nontumor samples 2.00 with an SD of 0.09. Difference between tumor and nontumor tissues was statistically significant (*P* < 0.0001), (Figure 3).

**Figure 4:**
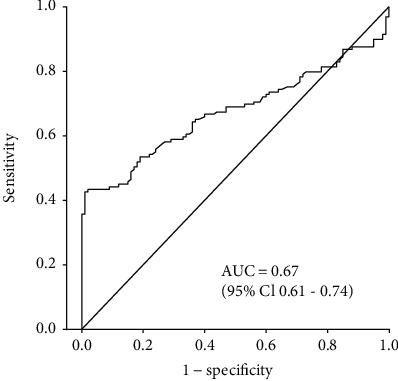
Receiver operating characteristic (ROC) analysis for *MYC* based on tumor and adjacent nontumor tissues from 129 lung cancer patients.

The ROC analysis revealed an AUC of 0.67 (95% CI: 0.61–0.74) (Figure 4). As the 95% CI does not include a value of 0.5 or less, the difference between the AUC and the default AUC of 0.5 is statistically significant.

Application of different cut-offs resulted in slightly different performances (Table 2). Using 95% specificity (cut‐off = 2.17) resulted in 43% sensitivity, whereas using the mean + 3 SD (cut‐off = 2.28) resulted in a lower sensitivity of 30% but a higher specificity of 100%. The cut-off of 2.19 based on the maximum YI resulted in 43% sensitivity and 99% specificity, representing 55 true-positive and one false-positive test.

*MYC* copy numbers were analyzed regarding clinicopathological parameters, showing no statistically significant association with age, sex, smoking status, histological subtype, grade, and stage (Table 3). In general, most samples within the different groups were *MYC* negative, i.e., the *MCY* copy number was <2.19. Notably, increased numbers of samples from patients with large-cell neuroendocrine carcinomas (LCNEC) and patients with stage T2 (six out of eight and 25 out of 45, respectively) were *MYC* positive (copy number ≥ 2.19), but differences were statistically not significant.

## 4. Discussion

*MYC* is a classical oncogene, and its amplification is a common event in lung cancer. In this study, we detected an increase of *MYC* copy numbers in 43% of the analyzed lung cancer samples. Published *MYC* alterations varied widely between 11% and 88% [8, 21]. The large range in published *MYC* alterations might be based on several factors, e.g., different study groups, different methods, and different cut-offs applied.

The performance of the *MYC* CNV assay utilizing dPCR is marked by a moderate sensitivity of 43% and a very high specificity of 99%, resulting in only one false positive test. In comparison, Flacco et al. revealed 61% sensitivity and 60% specificity using FISH (fluorescence in situ hybridization) for the detection of *MYC* copy numbers in a cohort of 113 NSCLC patients [22]. Applying a higher specificity of nearly 99% resulted in reduced sensitivity of approximately 20%. Interestingly, nearly all LCNEC were *MYC* positive, whereas barely half of the patients with other histological subtypes were *MYC* positive. Additionally, higher levels of *MYC* were observed in T2 tumors in contrast to T1 and T3 + T4 tumors. The cause for the increased *MYC* amplification in LCNEC and in T2 tumors remains unknown. To our knowledge, no significant differences were indicated for these groups in other studies [8, 10, 22]. However, the number of samples in the corresponding groups was low. Thus, these observations need to be verified in larger studies.

Common methods for the assessment of *MYC* CNV in tissues were FISH, CISH (chromogenic in situ hybridization) [23, 24], CGH (comparative genome hybridization) [25, 26], and qPCR [8, 27]. However, in contrast to dPCR, FISH, CSIH, and CGH are expensive and time-consuming methods, depending on subjective assessment [15, 28]. In comparison to qPCR, dPCR is marked by higher accuracy and reproducibility [24] and there is no need for a standard curve or calibrator sample within an analysis. Thus, dPCR might be a user-friendly method for the application in clinical diagnostics.

dPCR for the determination of *MYC* copy numbers has been performed only in a small number of studies for different tumor entities [28]. To our knowledge, analysis of *MYC* CNV in lung cancer using dPCR has not been described before. We show that the determination of *MYC* copy numbers using dPCR and qPCR revealed comparable results. Similarly, Yong et al. indicated a high correlation of dPCR and qPCR analyzing *AMY2A* (amylase alpha 2A) and *AMY1* (amylase 1) in whole blood from obese and nonobese subjects [29]. Additional, Shoda et al. demonstrated a correlation between dPCR and qPCR analyzing *HER2* (human epidermal growth factor receptor 2) in plasma of gastric cancer patients [30]. Thus, dPCR appears to be a reliable tool for the analysis of copy numbers [18].

A pretreatment of the gDNA prior to dPCR was suggested in order to improve the performance [31, 32]. For the analysis of *MYC* copy numbers, we used untreated as well as pretreated (digested and preamplified) gDNA. Basically, no differences were revealed for *MYC* copy numbers between treated and nontreated gDNA. However, differences could be observed for samples with *MYC* copy numbers > 3. As differences occur only for copy numbers above, the cut-off of 2.19 and the quantitation of the exact copy number might not be necessary; at least for the analysis of the *MYC* status using the described method, a pretreatment of the gDNA is not required. In agreement with our results, several studies showed that pretreatment did not improve the performance of copy number detection [28, 33–35]. This might be based on already fragmented gDNA or low gDNA concentration (<3 ng/*μ*L) in the samples [32].

It was suggested that lung cancer is marked by increased *MYC* copy numbers showing more aggressive phenotypes. In addition, it was indicated that *MYC* amplification was correlated with poor prognosis in small-cell lung carcinomas [9], NSCLC [22], and adenocarcinomas [8]. Unfortunately, no information was available regarding the survival time of the patients in this study.

Nevertheless, we confirmed that *MYC* amplification is a frequent event in lung cancer patients and may have high potential in clinical diagnostics for patient selection regarding personalized therapies. As Cappuzzo et al. reported that patients with amplified *MYC* were more sensitive to treatments with TKIs [10], the precise and fast assessment of the *MYC* status using dPCR might improve and speed up the decision trees in clinical routine.

A limitation of this study is the small number of samples in the analyzed subgroups, e.g., LCNEC as well as stages T3 and T4, because higher numbers would enable more meaningful analyses. Unfortunately, no information regarding the survival of the patients analyzed in this work was available, although the use of *MYC* as a prognostic marker might be interesting.

## 5. Conclusion

In conclusion, we demonstrated that dPCR is an accurate and reliable method for the assessment of the *MYC* status in tissues, showing that *MYC* amplification is a common event in lung cancer patients. The performance of the *MYC* copy number determination using a fixed high specificity of 99% is marked by a moderate sensitivity of 43%. Assessment of *MYC* status by dPCR might be useful in clinical diagnostics as a prognostic marker and for selecting an appropriate therapy. A future goal would be the development of a blood-based dPCR assay to determine the *MYC* status minimal invasively.

## Figures and Tables

**Table 1:** Patients' characteristics and clinicopathological parameters of the study group (*N* = 129).

Characteristic	Group	*N* (%)
Age (years), median (range)		67 (39-84)
Sex	Male	80 (62.0)
	Female	49 (38.0)
Smoking status	Never	15 (11.6)
	Former	70 (54.3)
	Current	44 (34.1)
Histological subtypes	Adenocarcinoma	67 (51.9)
	Squamous cell carcinoma	30 (23.3)
	Carcinoid	14 (10.9)
	LCNEC^∗^	8 (6.2)
	Other^#^	10 (7.8)
Grade^§^	G1	11 (8.5)
	G2	50 (38.8)
	G3	47 (36.4)
	G4	8 (6.2)
	Unknown	13 (10.1)
T stage	T1	62 (48.1)
	T2	45 (34.9)
	T3	16 (12.4)
	T4	3 (2.3)
	Unknown	3 (2.3)

^∗^LCNEC: large-cell neuroendocrine carcinoma. ^#^Four adenosquamous carcinomas, two non-small-cell lung cancers, one non-small-cell lung cancer (not otherwise specified), two small-cell carcinomas, and one synovial sarcoma. ^§^Grade 1: well differentiated, low grade; grade 2: moderately differentiated, intermediate grade; grade 3: poorly differentiated, high grade; and grade 4: undifferentiated, high grade.

**Table 2:** Performance of the dPCR-based *MYC* CNV assay calculated for different cut-offs.

	Cut-off	True positive (*N*)	True negative (*N*)	False positive (*N*)	False negative (*N*)	Sensitivity (%)	Specificity (%)
95% specificity	2.17	56	123	6	73	43	95
Maximum Youden's index	2.19	55	128	1	74	43	99
Mean + 2 SD^∗^	2.19	55	128	1	74	43	99
Mean + 3 SD	2.28	38	129	0	91	30	100

^∗^SD: standard deviation.

**Table 3:** *MYC* copy numbers regarding the analyzed clinicopathological parameters (*N* = 129).

			Tumor	
Characteristic	Group	*MYC* ≥ 2.19 (*N* = 55, 42.6%)	*MYC* < 2.19 (*N* = 74, 57.4%)	*P* value (chi^2^ test)
Age, *N* (%)	≤67 years	31 (56.4)	34 (45.9)	0.242
	>67 years	24 (43.6)	40 (54.1)	
Sex, *N* (%)	Male	34 (61.8)	46 (62.2)	0.968
	Female	21 (38.2)	28 (37.8)	
Smoking status, *N* (%)	Never	7 (12.7)	8 (10.8)	0.929
	Former	29 (52.7)	41 (55.4)	
	Current	19 (34.6)	25 (33.8)	
Histological subtypes, *N* (%)	Adenocarcinoma	29 (52.7)	38 (51.4)	0.338^∗^
	Squamous cell carcinoma	12 (21.8)	18 (24.3)	
	Carcinoid	4 (7.3)	10 (13.5)	
	LCNEC^#^	6 (10.9)	2 (2.7)	
	Other^§^	4 (7.3)	6 (8.1)	
Grade, *N* (%)	Missing	5 (9.1)	8 (10.8)	0.441
	G1 + G2	23 (41.8)	38 (51.4)	
	G3 + G4	27 (49.1)	28 (37.8)	
T stage, *N* (%)	Missing	2 (3.6)	1 (1.4)	0.092^∗^
	T1	22 (40.0)	40 (54.0)	
	T2	25 (45.5)	20 (27.0)	
	T3 + T4	6 (10.9)	13 (17.6)	

^∗^Fisher's exact test. ^#^LCNEC: large-cell neuroendocrine carcinoma. ^§^Four adenosquamous carcinomas, two non-small-cell lung cancers, one non-small-cell lung cancer (not otherwise specified), two small-cell carcinomas, and one synovial sarcoma.

## Data Availability

The raw data supporting the conclusions of this article are included as Additional File 1.

## Supplementary Materials

Supplementary Materials Additional File 1 Table 1: levels of RNaseP (copies/*μ*L) and MYC (copies/*μ*L) by dPCR. Table 2: levels of RNaseP (Ct) and MYC (Ct) by qPCR. Table 3: levels of RNaseP (copies/*μ*L) and MYC (copies/*μ*L) by dPCR.
