# Supplementary material for: Digital PCR for the Analysis of MYC Copy Number Variation in Lung Cancer
Source: Dis Markers. 2020 Sep 19;2020:4176376. doi: 10.1155/2020/4176376 (PMC7525309; doi:10.1155/2020/4176376)
Supplement: Supplementary Materials — Additional File 1 Table 1: levels of RNaseP (copies/μL) and MYC (copies/μL) by dPCR. Table 2: levels of RNaseP (Ct) and MYC (Ct) by qPCR. Table 3: levels of RNaseP (copies/μL) and MYC (copies/μL) by dPCR. [file 4176376.f1.pdf]

Table 1. Levels of *RNaseP* (copies/µL) and *MYC* (copies/µL) by dPCR

Sample	**N**on-**T**umor/ **T**umor	Non-treated		Digested		Pre-amplified	
		RNaseP	*MYC*	*RNaseP*	*MYC*	*RNaseP*	*MYC*
003	NT	692	704	814	840	851	876
	T	539	1008	770	1423	740	1500
015	NT	527	518	975	945	591	573
	T	455	555	953	1181	525	668
026	NT	803	811	864	825	439	487
	T	736	741	963	943	790	771
028	NT	885	886	1020	1067	421	414
	T	578	976	699	1166	765	1176
033	NT	742	719	980	958	907	907
	T	828	924	859	973	369	412
042	NT	881	875	899	968	448	481
	T	745	1583	850	1657	571	1266
046	NT	686	697	1061	1057	847	897
	T	439	532	1209	1553	511	611
049	NT	391	391	419	410	467	452
	T	381	462	540	641	469	547

Table 2. Levels of *RNaseP* (Ct) and *MYC* (Ct) by qPCR

Sample	**N**on-**T**umor/**T**umor	*RNaseP*	*MYC*
001	NT	25,50	24,76
	T	25,79	24,77
002	NT	25,54	24,82
	T	27,23	26,30
003	NT	25,91	25,09
	T	26,25	24,79
004	NT	25,94	25,15
	T	25,79	24,68
005	NT	25,63	24,89
	T	25,91	25,17
006	NT	25,82	25,08
	T	25,68	24,96
007	NT	25,60	24,81
	T	25,74	24,87
008	NT	25,77	24,94
	T	25,70	24,61
009	NT	25,76	25,02
	T	25,41	24,64
010	NT	25,59	25,12
	T	25,85	24,90
011	NT	25,63	24,93
	T	25,57	24,90
012	NT	25,92	24,96
	T	25,70	24,88
013	NT	26,12	25,44
	T	25,90	24,81
014	NT	25,68	24,97
	T	25,83	25,01
015	NT	25,77	25,19
	T	25,74	24,85
016	NT	25,76	25,01
	T	26,17	25,33
017	NT	25,90	25,11
	T	25,99	25,04
018	NT	25,94	25,31
	T	25,78	25,09
019	NT	26,09	25,41
	T	26,81	24,88
020	NT	26,03	25,25
	T	26,22	25,49
021	NT	26,24	25,44
	T	26,24	25,44
022	NT	26,16	25,38
	T	26,10	25,22
023	NT	25,98	25,18
	T	25,75	24,97
024	NT	25,59	24,73
	T	26,19	25,11

025	NT	25,98	25,33
	T	26,13	25,41
026	NT	26,26	25,42
	T	26,13	25,27
027	NT	25,93	25,13
	T	25,85	25,23
028	NT	26,18	25,31
	T	26,40	25,02
029	NT	26,22	25,32
	T	25,88	25,03
030	NT	25,93	25,12
	T	26,07	25,33
031	NT	25,86	25,10
	T	25,86	25,12
032	NT	25,75	25,07
	T	26,38	25,66
033	NT	25,81	25,08
	T	26,15	25,26
034	NT	26,01	25,48
	T	25,22	24,47
035	NT	26,03	25,22
	T	25,80	25,03
036	NT	25,43	24,87
	T	26,16	25,50
037	NT	25,54	24,94
	T	25,85	25,19
038	NT	25,19	24,46
	T	25,29	24,63
039	NT	26,00	25,28
	T	25,20	24,78
040	NT	25,41	24,87
	T	26,85	26,12
041	NT	25,66	24,89
	T	25,72	24,88
042	NT	26,12	25,38
	T	25,70	24,16
043	NT	25,67	24,96
	T	25,77	24,79
044	NT	25,81	25,12
	T	25,93	25,17
045	NT	25,82	24,92
	T	25,74	25,19
046	NT	25,72	24,93
	T	26,35	25,36
047	NT	25,72	24,89
	T	25,83	25,07
048	NT	25,67	24,97
	T	25,33	24,02
049	NT	26,74	25,96
	T	26,76	25,86

050	NT	26,33	25,58
	T	26,02	24,91
051	NT	25,61	24,96
	T	26,29	24,75
052	NT	25,80	25,10
	T	24,57	23,77
053	NT	26,24	25,51
	T	26,01	24,98
054	NT	25,74	25,01
	T	25,75	24,60
055	NT	25,60	25,00
	T	24,58	23,92
056	NT	25,97	25,23
	T	25,77	24,92
057	NT	25,81	25,11
	T	24,96	24,12
058	NT	26,40	25,68
	T	26,68	25,84

Table 3. Levels of *RNaseP* (copies/µL) and *MYC* (copies/µL) by dPCR

Sample	**N**on-**T**umor/**T**umor	*RNaseP*	*MYC*
001	NT	994	972
	T	789	977
002	NT	837	906
	T	544	561
003	NT	692	704
	T	539	1008
004	NT	520	486
	T	947	863
005	NT	691	692
	T	589	590
006	NT	981	956
	T	1155	1187
007	NT	853	893
	T	817	910
008	NT	762	785
	T	752	1078
009	NT	743	712
	T	725	723
010	NT	703	634
	T	719	957
011	NT	889	889
	T	867	840
012	NT	799	859
	T	1063	1204
013	NT	494	473
	T	432	529
014	NT	362	344
	T	432	529
015	NT	527	518
	T	455	555
016	NT	1720	1725
	T	975	981
017	NT	945	913
	T	611	697
018	NT	722	700
	T	748	753
019	NT	649	636
	T	356	798
020	NT	693	693
	T	500	488
021	NT	501	492
	T	536	531
022	NT	684	678
	T	549	570
023	NT	660	642
	T	868	868
024	NT	1001	1024
	T	557	652

025	NT	706	647
	T	664	619
026	NT	803	811
	T	736	741
027	NT	621	635
	T	826	736
028	NT	885	886
	T	578	976
029	NT	441	494
	T	696	797
030	NT	738	717
	T	713	720
031	NT	728	737
	T	814	826
032	NT	917	856
	T	977	934
033	NT	742	719
	T	828	924
034	NT	490	498
	T	1004	961
035	NT	746	738
	T	832	819
036	NT	1086	965
	T	613	637
037	NT	908	976
	T	702	598
038	NT	761	828
	T	1021	895
039	NT	620	618
	T	1274	1024
040	NT	888	854
	T	569	545
041	NT	997	958
	T	739	847
042	NT	881	875
	T	745	1583
043	NT	814	802
	T	614	769
044	NT	917	884
	T	786	783
045	NT	873	903
	T	843	883
046	NT	686	697
	T	439	532
047	NT	766	763
	T	648	715
048	NT	819	820
	T	898	1473
049	NT	391	391
	T	381	462

050	NT	553	551
	T	588	738
051	NT	764	740
	T	457	892
052	NT	783	751
	T	1155	1314
053	NT	582	565
	T	719	910
054	NT	834	819
	T	853	1369
055	NT	605	566
	T	1602	1561
056	NT	647	648
	T	812	786
057	NT	692	717
	T	1154	1230
058	NT	499	517
	T	399	436
059	NT	635	639
	T	645	709
060	NT	518	478
	T	679	856
061	NT	576	587
	T	434	456
062	NT	545	574
	T	763	792
063	NT	350	371
	T	500	880
064	NT	664	699
	T	818	895
065	NT	445	451
	T	657	748
066	NT	507	423
	T	572	712
067	NT	501	542
	T	552	528
068	NT	646	684
	T	1634	1703
069	NT	747	757
	T	517	597
070	NT	430	464
	T	556	536
071	NT	579	551
	T	625	638
072	NT	590	589
	T	684	648
073	NT	865	869
	T	854	892
074	NT	658	661
	T	620	627

075	NT	1063	1065
	T	611	603
076	NT	667	656
	T	593	759
077	NT	690	713
	T	528	536
078	NT	674	649
	T	558	565
079	NT	560	572
	T	542	461
080	NT	698	672
	T	753	702
081	NT	671	646
	T	773	714
082	NT	667	665
	T	690	1123
083	NT	738	695
	T	547	524
084	NT	719	724
	T	749	729
085	NT	663	628
	T	717	835
086	NT	596	593
	T	497	479
087	NT	794	770
	T	711	898
088	NT	792	782
	T	691	1004
089	NT	627	583
	T	582	582
090	NT	551	530
	T	673	836
091	NT	558	551
	T	539	611
092	NT	547	550
	T	573	602
093	NT	666	629
	T	640	480
094	NT	650	600
	T	645	676
095	NT	652	650
	T	482	468
096	NT	607	593
	T	667	654
097	NT	857	839
	T	686	709
098	NT	967	919
	T	698	510
099	NT	681	670
	T	628	677

100	NT	869	819
	T	531	454
101	NT	869	815
	T	696	701
102	NT	910	982
	T	959	1006
103	NT	947	982
	T	871	1343
104	NT	1214	1315
	T	881	1697
105	NT	1309	1365
	T	1196	1400
106	NT	1301	1410
	T	822	867
107	NT	807	810
	T	1076	1061
108	NT	1707	1744
	T	898	780
109	NT	1937	2026
	T	1231	1399
110	NT	593	623
	T	1295	1330
111	NT	786	807
	T	734	815
112	NT	1069	1101
	T	1797	1848
113	NT	713	741
	T	1310	1517
114	NT	707	724
	T	1070	1260
115	NT	520	547
	T	669	790
116	NT	975	998
	T	1354	1368
117	NT	1252	1362
	T	718	883
118	NT	1085	1169
	T	1539	1298
119	NT	1449	1573
	T	314	351
120	NT	728	789
	T	623	712
121	NT	659	712
	T	784	1517
122	NT	978	928
	T	778	854
123	NT	713	711
	T	877	631
124	NT	1341	1315
	T	1451	1254

125	NT	1619	1621
	T	1457	1608
126	NT	1360	1318
	T	1238	1214
127	NT	790	782
	T	693	665
128	NT	650	637
	T	677	697
129	NT	741	723
	T	846	807
